# NEW REFERENCE PARAMETERS FOR BODY MASS INDEX IN CHILDREN AGED SIX TO TEN YEARS

**DOI:** 10.1590/1984-0462/2021/39/2019129

**Published:** 2020-08-03

**Authors:** Alex Sander Freitas, Marise Fagundes Silveira, José Jorge Francisco de Santana, Marcos Flávio Silveira Vasconcelos D’Ângelo, Desirée Sant’Ana Haikal, Renato Sobral Monteiro-Junior

**Affiliations:** a Universidade Estadual de Montes Claros, Montes Claros, MG, Brazil.

**Keywords:** Body mass index, Overweight, Obesity, Childhood obesity, Nutritional status, Índice de Massa Corporal, Sobrepeso, Obesidade, Obesidade infantil, Estado nutricional

## Abstract

**Objective:**

To determine new body mass index (BMI) reference values to classify the nutritional status of children aged six to ten years old from the city of Montes Claros (state of Minas Gerais), Southeast Brazil.

**Methods:**

The sample consisted of 3,863 individuals from both genders. Body mass and height were measured to determine the BMI. We adopted the Lambda, Mu, and Sigma (LMS) method to obtain the cut-off points. After that, each stratum curve was smoothed using quartic polynomials by gender. Average interpolation was used to determine the biannual distribution values. We calculated the 3^rd^, 85^th^, and 95^th^ centiles to classify underweight, overweight, and obesity, respectively, according to gender and age.

**Results:**

After tabulating the LMS parameters at biannual intervals by gender, we plotted a graphic with seven centiles of BMI distribution and calculated the new BMI parameters for children aged 6-10 years old from the city of Montes Claros. The cut-off values for underweight, overweight, and obesity classification were, respectively, 17.5, 25 and 30 kg/m^2^.

**Conclusions:**

For the studied children, the use of traditional BMI references may result in the overestimation of underweight and underestimation of overweight and obesity. Studies should be carried out with periodic updates, respecting the characteristics of each location in order to use BMI reference values to classify the nutritional status of children and adolescents.

## RESUMO

**Objetivo:**

Determinar novos valores de referência do índice de massa corporal (IMC) para a classificação do estado nutricional de crianças de seis a 10 anos da cidade de Montes Claros (MG) Brasil.

**Métodos:**

Foi utilizada uma amostra de 3.863 sujeitos de ambos os sexos, sendo mensurados massa corporal e estatura para a determinação do IMC. Para a obtenção dos pontos de corte utilizou-se o método Lambda, Sigma e Mu (LMS). Em seguida, as curvas de cada estrato foram suavizadas por polinômios de 4º grau por sexo, e, por interpolação das médias aritméticas, foram obtidos os valores semestrais da distribuição. Foram calculados os percentis 3%, 85% e 95% para a classificação de baixo peso, sobrepeso e obesidade, respectivamente, de acordo com sexo e idade.

**Resultados:**

Após a tabulação em intervalos semestrais dos valores dos parâmetros LMS por sexo, foi possível construir o gráfico com sete centis da distribuição do IMC, sendo apresentados os novos parâmetros do IMC para crianças de 6 a 10 anos de Montes Claros, equivalentes aos valores 17,5, 25 e 30 kg/m^2^ para baixo peso, sobrepeso e obesidade respectivamente.

**Conclusões:**

Em relação à população de Montes Claros, a utilização das tradicionais referências para o IMC pode resultar em uma superestimativa do baixo peso e subestimativa do sobrepeso e obesidade. O que se sugere é que, para o uso de valores de referência do IMC para classificar o estado nutricional de crianças e adolescentes, sejam realizados estudos com atualizações periódicas, respeitando as características de cada localidade.

## INTRODUCTION

Obesity is considered one of the main public health problems worldwide, reaching increasing proportions regardless of social class, gender, and age group.^1^ Previous estimates indicated that the obese population would reach 1.5 billion people in 2012, with prospects for growth over time around the world.^2^ ^,^ ^3^ Studies reveal that obesity practically doubled globally between 1980 and 2008, and that between 1980 and 2013 the proportion of adults with body mass index (BMI) corresponding to 25 kg/m^2^ or more increased from 29 to 37% among men and from 30 to 38% among women.^4^ ^,^ ^5^

In general, the prevalence of obesity in Brazil increased by an average of 53% from the 1970s to the late 1990s and, in some Brazilian capitals, the proportion of overweight people grew from 42.7 to 46.6% between 2006 and 2009, while the number of obese individuals rose from 11.4 to 13.9%.^6^ ^,^ ^7^ In this context, childhood obesity has grown exponentially in most of the world, including Brazil.^8^ Data from the 2008-2009 Household Budget Survey indicate that 33.5% of children aged five to nine years were overweight, while 14.3% were obese.^9^

In addition to the increased risk of developing chronic diseases associated with overweight, such as high blood pressure, type 2 diabetes, orthopedic problems, and certain types of cancers, overweight children are likely to become obese adults and at greater risk of developing comorbidities.^10^ ^,^ ^11^

In view of this reality, several measures and reference values are being used for screening childhood overweight and obesity. In this regard, BMI remains a consensus for the classification of body weight ratios in the general population, as it presents high correlation with the current methods for assessing body capacity and is a simple measure, reducing financial costs and being applicable to population studies.^1^

However, BMI displays significant variation between age and gender in childhood and adolescence, requiring the use of specific cut-off points according to these variables. Consequently, different BMI classification criteria have been developed and implemented worldwide, including in Brazil.^1^ ^,^ ^11^

Considering that Brazil is a continental-size country with distinct geographic features, marked by ethnic and cultural plurality, as well as great socioeconomic inequality, and that Montes Claros, a Northern Minas Gerais city located in the Southeast Region of Brazil (the most developed in the country), presents socioeconomic indicators closer to those of less favored regions (North and Northeast),^12^ it is possible that the BMI reference values for the child population of this city might be different from those adopted nationally and internationally. In this scenario, the present study aims to determine new BMI reference values to classify the nutritional status of children aged six to 10 years in the city of Montes Claros, Minas Gerais, Brazil.

## METHOD

This is a one-arm cross-sectional study with quantitative data analysis. We followed the Strengthening the Reporting of Observational Studies in Epidemiology (STROBE) standards.^13^

Data were collected from 16 schools (ten public and six private) located in the city of Montes Claros, from February to December 2014. School selection and recruitment occurred by random drawing from a numbered list.

Prior to project approval by the Human Research Ethics Committee, we sent a letter of clarification along with an application for authorization to the Municipal Secretariat of Education of Montes Claros to obtain permission to visit the selected schools. Subsequently, a letter with the same content was delivered to the principal of each school to ask for authorization to carry out the research. In addition, those responsible for the selected children signed the informed consent form (ICF), allowing their participation in the study sample. School visits and data collection always occurred during Physical Education classes.

The selected students were properly enrolled and met the inclusion (age ranging from six to 9.9 years and a filled and signed ICF) and exclusion (participated in physical activities prior to data collection) criteria.

The sample was determined by stratification of the schoolchildren from a total of 81,088 students, from elementary to high school, resulting in 30,625 individuals in the group aged six to 9.9 years in the first elementary school grades. The sampling process was carried out according to clusters of the following school types: public (n=155) and private (n=93), totaling 248 institutions. Seventy schoolchildren from each group were randomly selected, 35 of each gender out of 16 schools, also randomly selected.

The sample size was established with a three-percentage-point error, a 95% confidence interval, and a 1.5 design effect (deff), with an increment of 10% for possible losses and/or refusals. Thus, 4,480 children were selected, of whom 329 were excluded due to the non-delivery of the signed ICF and/or absence during data collection. Therefore, the sample consisted of 4,151 students, 1,654 from private schools and 2,497 from public schools.

In addition to the authorization from the children’s guardians, all participants were informed about the procedures and objectives of the study, complying with Resolution No. 466/12 of the National Health Council and according to opinion No. 798,138 of the Research Ethics Committee of the Universidade Estadual de Montes Claros - UNIMONTES.

Anthropometric variables were determined according to Lohman, Roche, and Martorell.^14^ A digital scale with 0.1 kg precision and a stadiometer with 0.1 cm precision were used for data collection.

- **Body mass:** although body mass should be preferably measured with the subjects without clothing, we decided to restrict the dressing to light clothes, with the participants wearing bathing suits or shorts, short sleeve shirts, and barefoot.
- **Height:** the studied individuals, wearing the clothing mentioned above, stood close to the stadiometer, with the head adjusted by the researcher according to the Frankfurt Horizontal Plane.^14^
- **BMI:** this index was calculated by dividing the body mass (in kilograms) by the height (in meters) squared:^14^ body mass/height^2^.

We obtained the critical BMI values for the 6- to 10-year-old population from the city of Montes Claros by adopting the same method used to construct the international BMI standard proposed by the International Obesity Task Force (IOTF).^15^

We followed the LMS method, which consists of the Box-Cox transformation of positive independent data values to lead to a normal distribution. The sample was divided by gender and age group with cut-off points every six months, and BMI values lower or higher than two standard deviations from the mean of each group were excluded, preserving the number of 100 or more individuals, which is the minimum number suitable for the LMS method.^16^ The unconventional value of ±2 deviations was chosen to preserve sample homogeneity to the maximum.^17^ Thus, the sample underwent a reduction of 288 subjects, totaling 3,863 participants stratified by gender and age.

We calculated the LMS parameters for each age group, with L representing the coefficient (Box-Cox) used to transform BMI values to obtain a normal data distribution for each stratum. M indicates the median BMI value in each stratum, and S is the coefficient of variation of each stratum. The curves of each stratum were then smoothed by using quartic polynomials for each gender, and biannual distribution values were determined by average interpolation. After obtaining the three parameters, we constructed the curve for each centile using the formula proposed by Cole:^16^

$$C100\alpha (t)=M(t)\;\left[1+L(t)S(t)Z\alpha \right]1/L(t)$$

In which Zα is the normal deviation for area α; C100α is the centile corresponding to Zα; t is the age in months; and L, M, S, and C100α indicate the values for each curve at age t. Z-values equivalent to the 3^rd^, 85^th^, and 95^th^ centiles were used to determine the nutrition status classification - underweight, overweight, and obesity, respectively.

Initially, the LMS method required the data to be entered into and analyzed by the Minitab 18.0^®^ software developed by Minitab Inc. (State College, Pennsylvania, USA), which provided the Box-Cox transformations, median, and coefficient of variation of each stratum. We used the SPSS 22.0^®^ software developed by IBM (Armonk, New York, USA) for sample characterization regarding minimum, maximum, mean, standard deviation, and centile values, and the MatLab^®^ software developed by MathWorks Inc. (Natick, Massachusetts, USA) for curve smoothing and construction.

## RESULTS

Although the sample initially comprised 4,151 subjects, the exclusion of some participants was necessary due to the significant BMI variability caused by biological factors. Thus, we removed values above or below two standard deviations. Nonetheless, the small number of excluded individuals evidences data quality and preserves representativeness in the city of Montes Claros.

**Figure 1:**
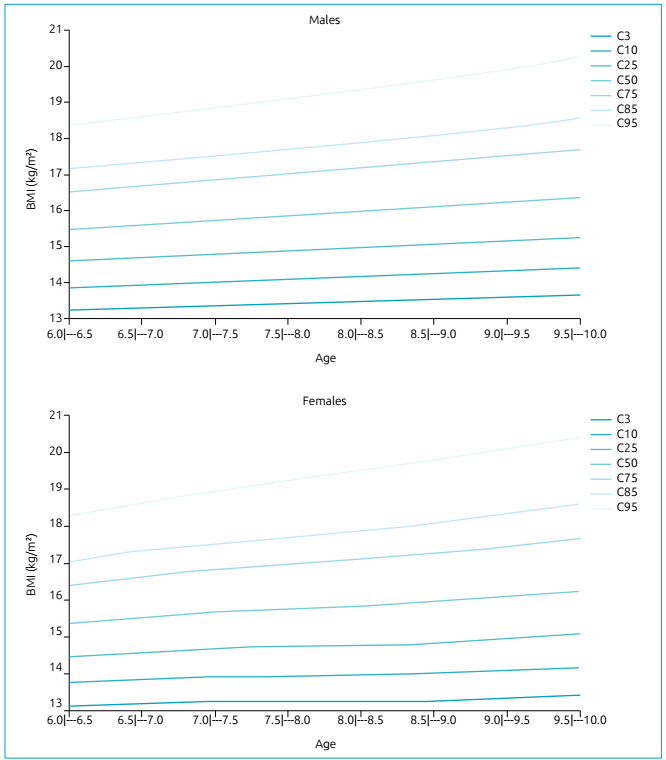
Centile distribution of body mass index curves among children (male and female) from Montes Claros - Minas Gerais.

The sample was then stratified by gender and age at 6-month intervals, resulting in a mean number by age group of 265 male (minimum of 197 and maximum of 330) and 253 female (minimum of 184 and maximum of 329) participants. Tables 1, 2, and 3 and Figure 1 present these values.

Table 1 shows descriptive BMI values. Increasing BMI values were associated with increasing age in both genders. Boys had slightly higher averages in comparison to girls.

**Table 1:** Descriptive body mass index (kg/m^2^) values according to gender and age group in children aged six to ten years from the city of Montes Claros - Minas Gerais.

Age (years)	Minimum	Maximum	Mean	Standard deviation
Males				
6.0 |--- 6.5 (n=243)	12.15	18.88	15.35	1.59
6.5 |--- 7.0 (n=261)	12.33	19.38	15.56	1.58
7.0 |--- 7.5 (n=237)	12.48	19.14	15.70	1.58
7.5 |--- 8.0 (n=206)	12.86	19.71	15.81	1.59
8.0 |--- 8.5 (n=251)	13.24	19.85	16.00	1.64
8.5 |--- 9.0 (n=278)	13.52	20.19	16.14	1.65
9.0 |--- 9.5 (n=301)	13.70	20.92	16.39	1.71
9.5 |--- 10.0 (n=187)	14.20	24.72	17.31	2.36
Females				
6.0 |--- 6.5 (n=201)	12.00	18.77	15.22	1.61
6.5 |--- 7.0 (n=306)	12.19	19.02	15.50	1.66
7.0 |--- 7.5 (n=236)	12.33	19.16	15.55	1.61
7.5 |--- 8.0 (n=221)	12.48	19.27	15.68	1.60
8.0 |--- 8.5 (n=224)	13.77	19.44	15.95	1.56
8.5 |--- 9.0 (n=238)	13.97	19.57	16.09	1.54
9.0 |--- 9.5 (n=294)	14.12	19.72	16.18	1.47
9.5 |--- 10.0 (n=179)	14.34	19.84	16.36	1.47

BMI: body mass index.

**Table 2:** LMS values for body mass index (kg/m^2^) distribution in the Montes Claros population aged six to ten according to gender and age group.

Age (years)	Males		
	L	M	S
6.0 |--- 6.5 (n=243)	-1.34	15.4978	0.0922
6.5 |--- 7.0 (n=261)	-1.41	15.6669	0.0933
7.0 |--- 7.5 (n=237)	-1.45	15.7206	0.0963
7.5 |--- 8.0 (n=206)	-1.47	15.8093	0.0991
8.0 |--- 8.5 (n=251)	-1.49	15.9507	0.1017
8.5 |--- 9.0 (n=278)	-1.51	16.0145	0.1050
9.0 |--- 9.5 (n=301)	-1.52	16.2252	0.1077
9.5 |--- 10.0 (n=187)	-1.48	16.3297	0.1107
Age (years)	Females		
	L	M	S
6.0 |--- 6.5 (n=201)	-1.38	15.3542	0.0942
6.5 |--- 7.0 (n=306)	-1.42	15.5713	0.0982
7.0 |--- 7.5 (n=236)	-1.45	15.6670	0.1009
7.5 |--- 8.0 (n=221)	-1.47	15.7769	0.1048
8.0 |--- 8.5 (n=224)	-1.49	15.8310	0.1085
8.5 |--- 9.0 (n=238)	-1.48	15.9550	0.1122
9.0 |--- 9.5 (n=294)	-1.46	16.0466	0.1156
9.5 |--- 10.0 (n=179)	-1.43	16.2083	0.1195

**Table 3:** Body mass index (kg/m^2^) values as a criterion for underweight, overweight, and obesity classifications in the population of Montes Claros aged six to ten years according to gender and age.

Age (years)	Males		
	UW	OW	OB
6.0 |--- 6.5 (n=243)	13.26	17.17	18.37
6.5 |--- 7.0 (n=261)	13.39	17.39	18.64
7.0 |--- 7.5 (n=237)	13.39	17.52	18.83
7.5 |--- 8.0 (n=206)	13.41	17.68	19.06
8.0 |--- 8.5 (n=251)	13.48	17.90	19.35
8.5 |--- 9.0 (n=278)	13.47	18.04	18.58
9.0 |--- 9.5 (n=301)	13.60	18.35	19.96
9.5 |--- 10.0 (n=187)	13.62	18.53	20.21
Age (years)	Females		
	UW	OW	OB
6.0 |--- 6.5 (n=201)	13.10	17.06	18.29
6.5 |--- 7.0 (n=306)	13.22	17.39	18.72
7.0 |--- 7.5 (n=236)	13.25	17.56	18.96
7.5 |--- 8.0 (n=221)	13.27	17.77	19.26
8.0 |--- 8.5 (n=224)	13.25	17.91	19.50
8.5 |--- 9.0 (n=238)	13.28	18.14	18.81
9.0 |--- 9.5 (n=294)	13.29	18.32	20.07
9.5 |--- 10.0 (n=179)	13.34	18.59	20.43

UW: underweight; OW: overweight; OB: obesity.

Table 2 indicates the LMS values used to obtain the new BMIs for nutritional status classification.

Table 3 presents the BMI according to gender and age with three classification parameters. The 3^rd^ centile corresponded to underweight individuals and the 85^th^ centile to overweight ones. Thus, all participants with BMI between the 3^rd^ and 85^th^ centiles were classified as having a normal weight. The 95^th^ centile indicates obesity. Figure 1 presents the BMI centile curve behavior in each age group by gender.

## DISCUSSION

The present study identified that critical BMI values for children aged six to ten years in the city of Montes Claros, as well as their respective classification, differ from those established by international and national guidelines. This situation may also occur in other regions, probably due to the diversity of geographic, cultural, and even biological characteristics of individuals.

BMI is routinely used as a tool to monitor the developmental process of children, as well as classify their nutritional status.^18^ ^,^ ^19^ On the other hand, it is noteworthy that BMI is affected by the developmental process dynamics and, as such, should not be used in the same way as in adults. In addition, BMI in children should be interpreted with caution, as it not necessarily indicates excess body fat, with body composition measurements being more sensitive in this regard.^18^ ^,^ ^19^

The BMI reference values are considered an adequate tool to classify the nutritional status of children and adolescents. However, statistical procedures that provide support and robustness are required so that these values display specificity according to the reference population.

Therefore, using the LMS method proposed by Cole et al.^16^ seems sufficient to construct tables with specific BMI reference values according to gender and age. Thus, the study, using a final sample of 3,863 schoolchildren aged six to 10 years from both genders, proposed the establishment of new reference values for the Montes Claros population through the mentioned method.

Katzmarzyk et al.^20^ point out that childhood obesity results from a complex interaction of multiple factors over time and that the contribution of each of these factors to obesity may not be the same in different regions around the globe. Various levels of influence, such as local and national policies, and numerous culturally-specific behavioral configurations affect such phenomena. This argument justifies the use of reference BMI values or other anthropometric indices specific to each region for nutritional status classification.

Assessments concerning the somatic growth process determined by anthropometric indices are essential, especially in relation to nutritional status monitoring and identification of possible changes in the health status of the pediatric population. In this regard, reference values aid in evaluating both growth and nutritional status, such as overweight and obesity in children and adolescents. Nevertheless, the growth pattern changes through time, and regular updates on these reference values are necessary.^21^

Over the last decades, several methodologies have been developed and implemented to establish cut-off points of anthropometric indices for children and adolescents, in order to highlight a certain growth pattern and nutritional status in pediatric populations from different countries.^21^ ^,^ ^22^ Among them, the LMS method presented by Cole & Green^23^ is a robust statistical procedure using centile values for anthropometric variables that are not normally distributed in the population.^22^

The LMS method consists of three parameters, Lambda (L), Mu (M), and Sigma (S), with L corresponding to the Box-Cox power lambda, M to the median of the values presented in a given group, and S to the coefficient of variation. The International Obesity Task Force (IOTF)^15^ also adopted this methodology to propose reference values for BMI classification in children and adolescents, and Conde and Monteiro^22^ used it to determine BMI cut-off points to assess the nutritional status of Brazilian children and adolescents.^17^ ^,^ ^21^

The BMI values presented herein for each gender and age range are different from the cut-off points established in other studies that propose to determine a parameter for the classification of nutritional status of children and adolescents. These differences are present in several studies that aimed to compare the performances and specificities of reference values established by the Center for Disease Control and Prevention (CDC),^24^ IOTF,^15^ Conde and Monteiro,^17^ World Health Organization (WHO),^25^ among others.^19^ ^,^ ^26^

Differences between the proposals become evident when directly comparing the values found in the present study with those reported by national and international classification systems. In the case of the investigation carried out by Conde and Monteiro,^17^ which establishes BMI classification values for Brazilian children and adolescents, most underweight values are lower than those presented in this research, while overweight and obesity values are higher.

Regarding the cut-off points proposed by the IOTF,^15^ all data on overweight and obesity referring to the same age groups of both genders are higher than those suggested herein for the population of Montes Claros - Minas Gerais.

Compared to the CDC,^24^ the values for girls aged six to 10 years from Montes Claros are all lower for both overweight and obesity. Among boys, we found a variation: in those aged up to 7.5 years, the cut-off points for overweight are higher in children from Montes Claros, while all CDC values are higher for obesity.^24^

Leal et al.^27^ tested and compared the specificity of BMI classification systems for children and adolescents according to the values proposed by the WHO,^25^ IOTF,^15^ and Conde and Monteiro.^17^ The authors used a sample of children aged seven to 10 years from Santa Catarina, Brazil, and identified that all approaches are effective in the screening of childhood overweight and obesity. However, the Brazilian reference pointed to a greater balance in the screening of overweight individuals, suggesting that regional aspects could explain the better performance of the values proposed by Conde and Monteiro.^17^

In contrast, Oliveira et al.^28^ revealed that the cut-off points recommended by the WHO^25^ are more sensitive for obesity identification compared to other criteria, such as those stipulated by the CDC^24^ and the National Center for Health Statistics (NCHS).^29^ In this case, we emphasize that the WHO proposal was revised and updated in 2007,^25^ demonstrating the need for periodic updates concerning reference values.

Moreover, Kêkê et al.^19^ compared the BMI of French children and adolescents aged four to 12 years to references from the WHO,^25^ IOTF,^15^ and the French proposal for BMI classification for children and adolescents. Overall, WHO^25^ values led to an overestimation of overweight and/or obesity compared to the French classification and the IOTF.^15^ However, the association between references depends on age groups and gender. In addition, the French reference values seem to agree closely with those from IOTF^15^ with respect to the overweight definition, especially in children aged seven to 12 years.

The variations observed in different classification criteria of the nutritional status of children and adolescents, caused by using BMI in distinct populations and periods, reflect the need to consider probable misunderstandings regarding the obtained results, underestimating or overestimating a given condition. The use of values that do not meet the actual needs of a determined region may, ultimately, result in inadequate public health strategies.^19^

Therefore, we should take into account that children’s growth patterns are subject to changes according to aspects related to time, region, ecology, environment, and genetics. In this respect, reference values should specifically fit a given area and be continuously updated. Furthermore, curves should be specially adjusted for the population of interest.^21^ Nonetheless, we underline that, even with this variety of BMI cut-off criteria for the classification of the nutritional status of the pediatric population, the Brazilian Society of Pediatrics (*Sociedade Brasileira de Pediatria* - SBP) uses and recommends that health professionals use the reference curves proposed by the WHO.^30^

We highlight the limitations of the present study, particularly regarding values restricted to a small age range, with further studies being necessary to cover the entire period that includes childhood and adolescent years. Consequently, we suggest that new studies be carried out and that reference values to classify the nutritional status of children and adolescents using BMI should be adopted according to a temporal logic, with periodic updates and within a regional specificity, respecting the characteristics of each location.

In conclusion, the values presented herein to classify the nutritional status of children aged six to ten years in the population of Montes Claros, Minas Gerais, do not agree with those available in the literature and most commonly used by the scientific community. As to the underweight classification, the present study reports values higher than those found in other references. On the other hand, the overweight and obesity values of this research are lower when compared to other studies. These contradictions raise an important concern regarding misguided strategies, and may even mislead experts into wrongful diagnoses. Hence, further studies should be conducted in other Brazilian regions, allowing a better understanding of BMI uses in children and adolescents.
